# Epidemic Cholera

**Published:** 1866-10

**Authors:** R. Dexter

**Affiliations:** Chicago


### CHICAGO MEDICAL EXAMINER.

N. S. DAVIS, M.D., Editor.

VOL. VII. OCTOBER, 1866. NO. 10.

### Original Contributions.

ARTICLE XXXV.

### EPIDEMIC CHOLERA.—A FEW REMARKS ON ITS PATHOLOGY AND TREATMENT.

By R. DEXTER, M.D., Chicago.

Owing to the double interest of an epidemic being upon us, and so many of its principles lying concealed in the mysteries of pathology, a few observations from almost any one may be acceptable.

As its great importance has engaged the best energies of many able and distinguished investigators of both continents, it might well seem presumptuous in me to hope to contribute anything worthy of the consideration of the profession, were it not that the constant progress of physiology and pathology is such as to open up new avenues for inquiry, and ever affording fresh facilities for pursuing them. This subject involves many facts which lie at the very foundation of these sciences, and many others which can only be deduced from analogy, while some remain, as yet, unexplained. The difficulties in its study do not seem to depend so much upon a want of observation, as upon its hidden primary cause and complicated actions. If we carefully trace the gradual digression from a truly physiological action through the entire changes of its pathological course, in a typical case of cholera, we will find about the following order of events:—

1. Slight derangement of alimentary canal, muscular languor and tremulousness, restlessness, headache, and anorexia.

2. An enhanced condition of the preceding phenomena, with abdominal uneasiness, perhaps actual vomiting and purging, tongue coated, cramping in distal and peripheral parts, arrest of secretion of urine, with coldness of surface.

3. Active vomiting, purging of the characteristic rice-water dejections, respiration labored, excessive, and painful, cramping of both body and extremities, great coldness of surface, and pulse variable.

4. Dyspnœa very great, peripheral circulation nearly suspended, venous congestion, labored cardiac action, skin livid, cold, and bathed in perspiration, great precardial pain, and coolness of respired air.

5. Low irritative fever, with an irritated and *congested condition* of one or more vital organs.

Many prominent men claim that the specific action of the cholera poison is upon the capillary circulation, some say pulmonary, others, general. To this I must, in part, disagree. That the capillary system is affected, is unquestionably correct; but it seems to me that there is as much evidence to confirm an opinion that its primary action is upon the nervous, glandular, or cellular systems, or that it acts, through the blood, upon any other system, as upon capillaries alone. Now, my belief is, that as soon as any poison has entered the blood, it acts upon its composition and changes its constituent elements; that it changes the constituent elements of the cells and their contents; that this modifies *absorption* and *secretion*, and thus alters *assimilation* and *excretion*.

That all known hæmatic poisons have some individual peculiarities, but many properties in common; and that all poisons taken into the blood must be eliminated by some of the emunctories; and that they have a specific predilection to escape from one or more eliminating systems, and when these are dormant that a compensating action is established, by which the other systems of emunctories eliminate what the disabled ones failed to expel. These principles are as well illustrated and as well settled, in regard to the elimination of poisons, as they are in the more ordinary every-day work of excretion.

I will here cite the action of antimony, and compare it with that of the cholera poison, assuming that they are both taken into the blood slowly, but gradually increased in amount, so that the antimony is received into the system about as cholera poison usually is, and their similarity in many respects will be very apparent. Their analogous effects are:—1. Prostration. 2. Irritation of the alimentary canal. 3. Impaired function of pneumogastric and other organic nerves. 4. Impoverishment of the blood. 5. Lowering of animal heat. 6. Excessive vomiting and purging, and, 7. Cold, clammy perspiration. Their differential actions are:—1. In cholera, there is a greater tendency to spasms, causing an early suspension of function, and thus preventing the elimination of its poison. 2. A stronger tendency to recession of capillary fluids to internal organs, and this want of fluid in the capillaries results in an endosmatic action from the surrounding tissues. 3. This overloading of larger veins forces the watery portion of the blood through into the bowels, and, hence, the *rice-water dejections.* That there is, in cholera, an early arrest of functional and capillary action, and, consequently, of elimination, is proved by the following conditions:—

1. An arrest of secretions, *e.g.*, urine and bile.
2. A gradual subsidence of animal heat.
3. An imperfect aëration of the blood.
4. Undue fulness of larger vessels.
5. Conditions of nutrition and disintegration in those who recover from its first stages.
6. Excessive nervous irritation.

Judging from the above facts, it seems to me that the indications for treatment are as patent as are the principles of medicine. They are to restore the action of the emunctories by recalling the receding capillary circulation. If this is accomplished, nature herself will be adequate to the task of elimination and restoration. The truth of the foregoing statements has been abundantly verified in our own practice. The above indications may be fulfilled by:—

1. Allaying nervous irritation.
2. By artificially restoring animal heat.
3. By a proper administration of the true eliminating medicines.

Nervous irritation is probably best allayed by opium in regular and sustained stimulant doses, until capillary action is provoked. Perhaps the preferable preparations are the tinct. and camph. tinct. opium, with such modifications as the case requires; if the discharges are excessive, with injections of plumbi acet. and morph. acet. The best artificial method of restoring animal heat, is to completely envelope the patient (excepting his face) in wet blankets, as hot as can be tolerated, and these hot envelopes are to be changed as often as their temperature is appreciably diminished. We should, also, (if the case proves in any way obstinate,) strictly enjoin recumbent rest, with sinapisms to the epigastrium and præcordia, and administer ice and ice-cold water, to be swallowed every few minutes.

As soon as the excessive cramping, vomiting, and purging has subsided, administer the true eliminatives, those that will not only call into action the stomach and bowels, but the skin, lungs, kidneys, liver, absorbents, in fact, every conceivable method of judicious elimination.

My selection is usually a mercurial one, because it, *per se*, has the very effect we should produce—cathartic, catalytic, and eliminative. Camphor and ammonia, I find very useful. They materially aid pulmonary, cutaneous, and urinary elimination, and tone up the depressed pneumogastric and other organic nerves. In my practice, heat and moisture have overcome the congestion of the kidneys and liver better than anything else. I apply hot wet cloths to the lumbar and hypochondriac regions.

The above is a concise statement of my plan of treatment, with the principles upon which it is based, and the objects intended to be accomplished. I will here give notes of cases subject to this plan of treatment in my practice, with a success truly flattering, and one that the profession can repeat:—Of 78 cases, 63 were treated upon this method at the commencement of the cramping and rice-water dejections, and all recovered. Of the remaining 15, who were not seen until much later in the disease, (bordering upon collapse,) only 6 died.

For a further consideration of the elimination of hæmatic poison, the reader is referred to Dr. HEADLAND'S work on the Action of Medicine, chapter on eliminations; and Dr. WILLIAMS' Principles of Medicine, section on toxemia. The plan, so highly recommended by some authors, of allowing the emetic and cathartic action to continue, or even increasing it, whilst it has some show for elimination, seems to me to hasten collapse and cut off the chances for general emunctorial excretion; and we thus seem to get a pell-mell elimination instead of an elective one. Chloroform, I think, is contra-indicated by a highly carbonized condition of the blood, by an already embarrassed condition of the organic nerves, and by an oppressed condition of the heart and lungs. [See GROSS' System of Surgery, third edition, pages 539 and 540.]

In a state of collapse, when the watery portion of the blood has been drained away by the excessive evacuations, and where prostration marks every organ of the body, it is my opinion that little can be done, except by supplying such nourishment as requires but little digestion, and that will serve to sustain vital and recuperative action—beef-essence, fresh milk, coffee or tea, as the patient is accustomed, chicken broth, etc. Perhaps I ought here to mention some of my reasons for considering the rice-water dejections the result of pressure. They are: 1. The abdominal veins are congested, and with such congestion we always have an aqueous exudation. 2. Anatomically, this is the most free exudating surface. 3. Hydrogogue cathartics are probably exuded into the lower portion of the intestines, as a result of congestion. 4. Nervous shocks, as fear, cold, and the like, produce such action, especially when the kidneys are inactive, as is the case in cholera.

